# Modulation of Vascular Cell Function by Bim Expression

**DOI:** 10.1155/2013/297537

**Published:** 2013-10-31

**Authors:** Margaret E. Morrison, Tammy L. Palenski, Nasim Jamali, Nader Sheibani, Christine M. Sorenson

**Affiliations:** ^1^Department of Pediatrics, University of Wisconsin School of Medicine and Public Health, 600 Highland Avenue, H4/444 CSC, Madison, WI 53792-4108, USA; ^2^Department of Ophthalmology and Visual Sciences, University of Wisconsin School of Medicine and Public Health, Madison, WI 53792, USA; ^3^McPherson Eye Research Institute, University of Wisconsin School of Medicine and Public Health, Madison, WI 53792, USA

## Abstract

Apoptosis of vascular cells, including pericytes and endothelial cells, contributes to disease pathogenesis in which vascular rarefaction plays a central role. Bim is a proapoptotic protein that modulates not only apoptosis but also cellular functions such as migration and extracellular matrix (ECM) protein expression. Endothelial cells and pericytes each make a unique contribution to vascular formation and function although the details require further delineation. Here we set out to determine the cell autonomous impact of Bim expression on retinal endothelial cell and pericyte function using cells prepared from Bim deficient (Bim^−/−^) mice. Bim^−/−^ endothelial cells displayed an increased production of ECM proteins, proliferation, migration, adhesion, and VEGF expression but, a decreased eNOS expression and nitric oxide production. In contrast, pericyte proliferation decreased in the absence of Bim while migration, adhesion, and VEGF expression were increased. In addition, we demonstrated that the coculturing of either wild-type or Bim^−/−^ endothelial cells with Bim^−/−^ pericytes diminished their capillary morphogenesis. Thus, our data further emphasizes the importance of vascular cell autonomous regulatory mechanisms in modulation of vascular function.

## 1. Background

Apoptosis facilitates the removal of unwanted cells during development and maintains tissue homeostasis. Bcl-2 family members influence apoptosis in either a positive or a negative fashion. Family members are classically grouped into three subclasses including one that inhibits apoptosis, a second that induces apoptosis and a third contains that family members, such as Bim, that only have a BH3 domain that binds antiapoptotic family members to promote apoptosis [1]. Bcl-2 exerts opposing effects with regards to apoptosis compared with Bim, consistent with their opposing effects on cell adhesion and migration [2]. The removal of a single allele of Bim is sufficient to prevent the degenerative disorders caused by Bcl-2 deficiency [3, 4]. Bim expression is also essential for apoptosis of a wide range of growth factor deprived cells [5], including endothelial cells and pericytes [6]. Therefore, improper modulation of apoptosis could impact the development and/or the pathogenesis of many diseases including diabetic retinopathy.

Murine retinal vascular development proceeds after birth and is complete by postnatal day 21 (P21). Remodeling and pruning of the retinal vasculature continue until P42 [7, 8]. Pro- and anti-apoptotic factors regulate apoptosis during retinal vascular development, remodeling, and regression [8–10]. Bim influences not only apoptosis but also cell adhesion, migration, and extracellular matrix (ECM) protein expression. The ability of Bim to impact both apoptosis and the ECM milieu is integral to its role during retinal vessel remodeling, and regression. However, whether Bim expression impacts retinal endothelial cell and pericyte function in a similar manner remains to be determined.

Our previous studies demonstrated that Bim modulates retinal vascular development, remodeling, and regression [11]. Bim deficient (Bim^−/−^) mice demonstrated precocious formation of the retinal vascular plexus, increased vascular density, and attenuated hyaloid vessel regression. Loss of Bim expression also protected the retinal vasculature from hyperoxia-mediated vessel obliteration and neovascularization during oxygen induced ischemic retinopathy (OIR) [11]. These studies indicated that Bim expression is essential for retinal vascular remodeling but did not implicate a specific vascular cell type. Thus, understanding the role Bim plays in modulating endothelial cell and pericyte function will give us further insight into how aberrant regulation of retinal vessel development and remodeling can be avoided to preserve vision. 

 Endothelial cells and pericytes play specialized roles in the retina during the development and maintenance of the vasculature. Pericyte coverage of vascular sprouts stabilizes the vessels while endothelial cells provide the inner lining of the blood vessel [12, 13]. Retinal endothelial cell and pericyte dysfunction can lead to retinal vascular rarefaction and subsequent vision loss as the one that occurs in diabetic retinopathy. Here, we assessed whether the loss of the proapoptotic protein Bim differentially impacts the retinal endothelial cell and pericyte function. We observed an increased VEGF expression, ECM protein production, cell migration, proliferation, and adhesion in Bim^−/−^ retinal endothelial cells compared with wild-type cells. Although the decreased endothelial nitric oxide synthase (eNOS) expression corresponded with decreased nitric oxide (NO) production in Bim^−/−^ retinal endothelial cells, capillary morphogenesis was similar to that observed in wild-type retinal endothelial cells. In contrast to Bim^−/−^ retinal endothelial cells, Bim^−/−^ pericytes displayed decreased proliferation compared with their wild-type counterpart. Bim^−/−^ pericytes also demonstrated increased migration, adhesion, and VEGF expression. Coculturing wild-type or Bim^−/−^ retinal endothelial cells with Bim^−/−^ pericytes diminished capillary morphogenesis. The data presented here demonstrate that the deletion of Bim differentially impacts retinal endothelial cell and pericyte function, perhaps through the modulation of response to their microenvironment.

## 2. Results

### 2.1. Bim^−/−^ Retinal Endothelial Cells and Pericytes Demonstrate Aberrant Proliferation

**Figure 1:**
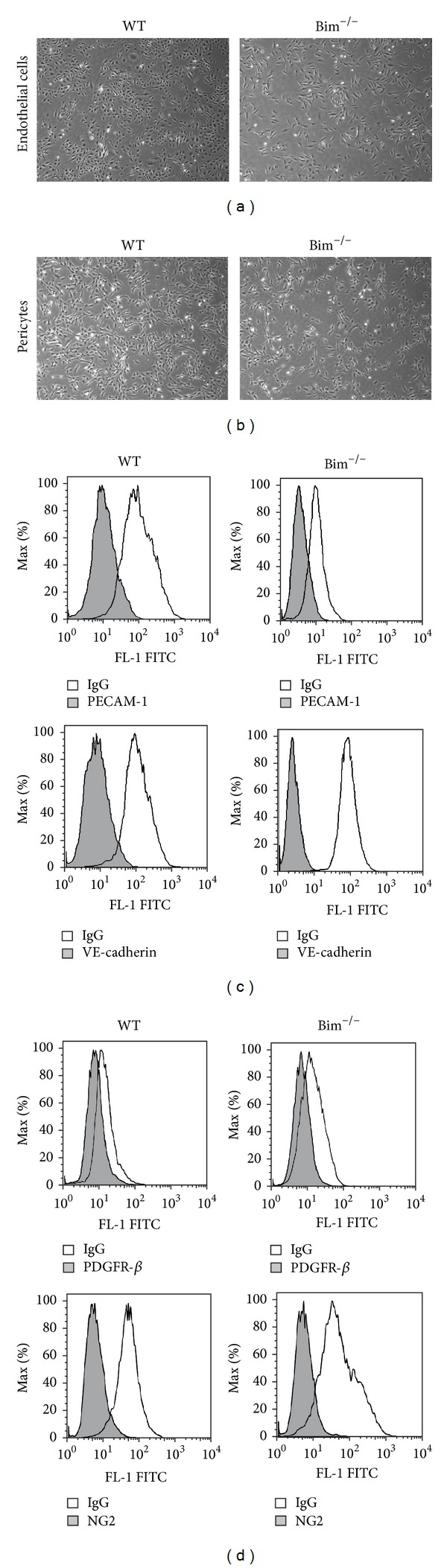
Wild-type and Bim^−/−^ cells morphology. Wild-type and Bim^−/−^ retinal endothelial cells (panel (a)) and pericytes (panel (b)) were cultured on gelatin or uncoated plates, respectively. Cells were photographed using a phase microscope in digital format at low magnification (×40). In panel (c), retinal endothelial cells prepared from wild-type and Bim^−/−^ mice were examined for the expression of PECAM-1 and VE-cadherin FACScan analysis. In panel (d), retinal pericytes from wild-type and Bim^−/−^ mice were examined for the expression of NG2 and PDGFR-*β* by FACScan analysis. The shaded areas show staining in the presence of control IgG.

To further investigate the role Bim plays in retinal vascular function, we isolated retinal endothelial cells and pericytes from wild-type and Bim^−/−^ mice as previously described [2, 14–17]. We first examined retinal endothelial cell and pericyte morphology and their expression of cell specific markers to confirm that these cells maintain their endothelial cell and pericyte characteristics. Bim^−/−^ retinal endothelial cells displayed a slightly elongated morphology compared to wild-type cells when plated on gelatin-coated plates (Figure 1(a)). The morphology of wild-type and Bim^−/−^ retinal pericytes was similar (Figure 1(b)). Wild-type and Bim^−/−^ retinal endothelial cells expressed the endothelial cell markers VE-cadherin and PECAM-1 (Figure 1(c)). Retinal pericytes expressed the pericyte markers platelet derived growth factor-receptor *β* (PDGFR*β*) and neuroglia proteoglycan 2 (NG2) (Figure 1(d)).

**Figure 2:**
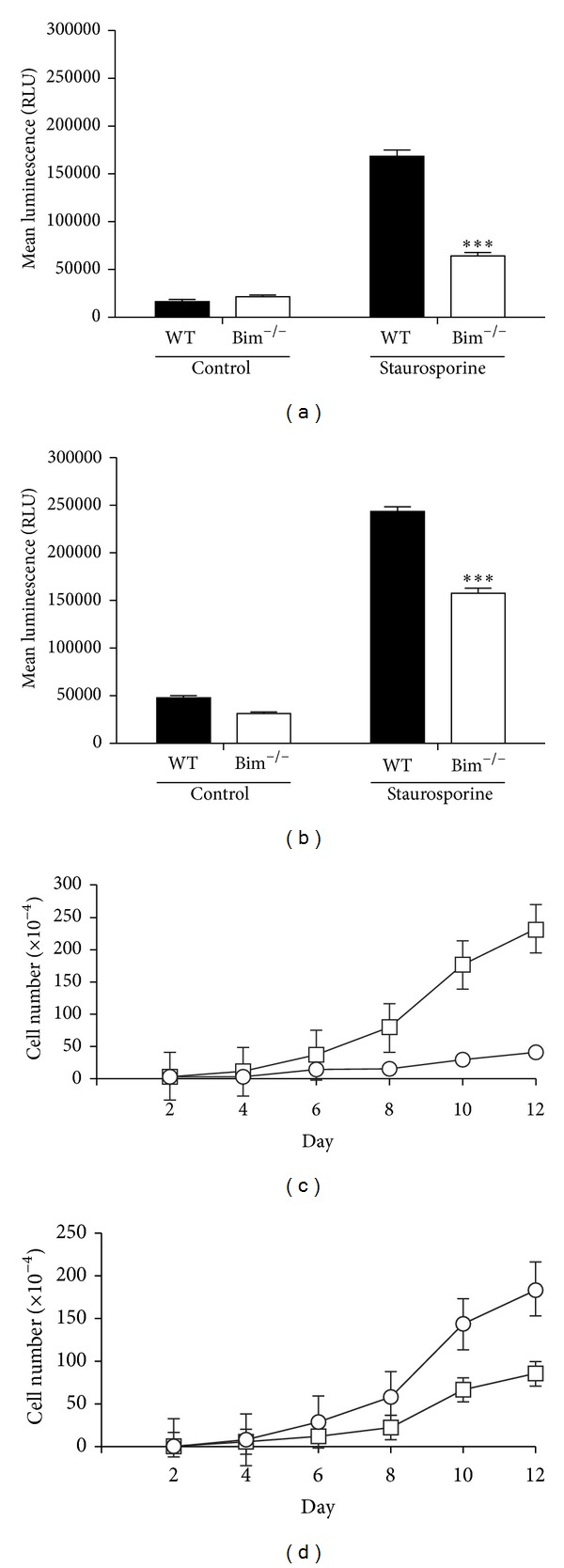
Modulation of proliferation in Bim^−/−^ cells. Wild-type and Bim^−/−^ retinal endothelial cells (panel (a)) and pericytes (panel (b)) were incubated with 10 nM staurosporine for 24 hours. Apoptosis was determined using a Caspase-Glo 3/7 assay. Wild-type and Bim^−/−^ retinal endothelial cells (panel (c)) and pericytes (panel (d)) were monitored for their growth rate over a two-week time frame for wild-type (○) and Bim^−/−^ (□) cells. These experiments were repeated twice with similar results (***P < 0.001).

Next we examined the rate of retinal endothelial cell and pericyte apoptosis and proliferation. Wild-type and Bim^−/−^ retinal endothelial cells and pericytes were incubated with staurosporine to induce apoptosis. Although the basal levels of apoptosis were similar, upon incubation with staurosporine for 24 h, Bim^−/−^ endothelial cells and pericytes demonstrated less apoptosis compared to wild-type cells (Figures 2(a) and 2(b)). Next cell proliferation was examined by counting the number of cells every other day for 2 weeks.  Figure 2(c) shows that Bim^−/−^ retinal endothelial cells proliferated at a faster rate than their wild-type counterpart. In contrast, Bim^−/−^ pericytes demonstrated a reduced rate of proliferation compared to their wild-type counterpart (Figure 2(d)).

### 2.2. Loss of Bim Expression Enhances Retinal Endothelial Cell and Pericyte Migration

**Figure 3:**
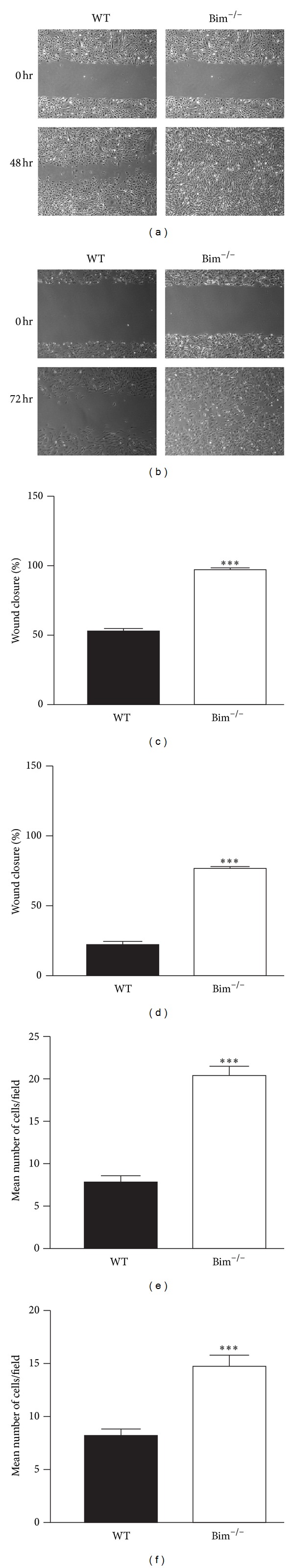
Bim^−/−^ retinal endothelial cells and pericytes demonstrate increased migration. Cell migration was determined by the scratch wounding of retinal endothelial cells (panel (a)) or pericytes (panel (b)) monolayers, and wound closure was monitored using a phase microscope in digital format at low magnification (×40). A representative experiment is shown here. Please note that wild-type vascular cells migrate slower than Bim^−/−^ cells. The quantitative assessment of the data is shown in panels (c) and (d). Transwell assays were performed with wild-type and Bim^−/−^ retinal endothelial cells (panel (e)) and pericytes (panel (f)). These experiments were repeated twice with similar results (***P < 0.001).

An appropriate rate of migration is essential for optimal capillary morphogenesis. Here we examined cell migration characteristics using scratch wound and transwell migration assays. A confluent monolayer of wild-type and Bim^−/−^ endothelial cells (Figure 3(a)) and pericytes (Figure 3(b)) was wounded, and 5-fluorouracil (5-FU) was added to prevent cell proliferation. Both Bim deficient retinal endothelial cells and pericytes migrated faster than their wild-type counterparts (Figures 3(a) and 3(b)). A quantitative assessment of the scratch wound data for endothelial cells and pericytes is shown in Figures 3(c) and 3(d), respectively. Enhanced migration of Bim^−/−^ endothelial cells and pericytes was also observed using a transwell migration assay as shown in Figures 3(e) and 3(f), respectively. Thus, the lack of Bim expression enhances the migration of retinal vascular cells.

### 2.3. Bim^−/−^ Retinal Vascular Cells Were More Adherent

**Figure 4:**
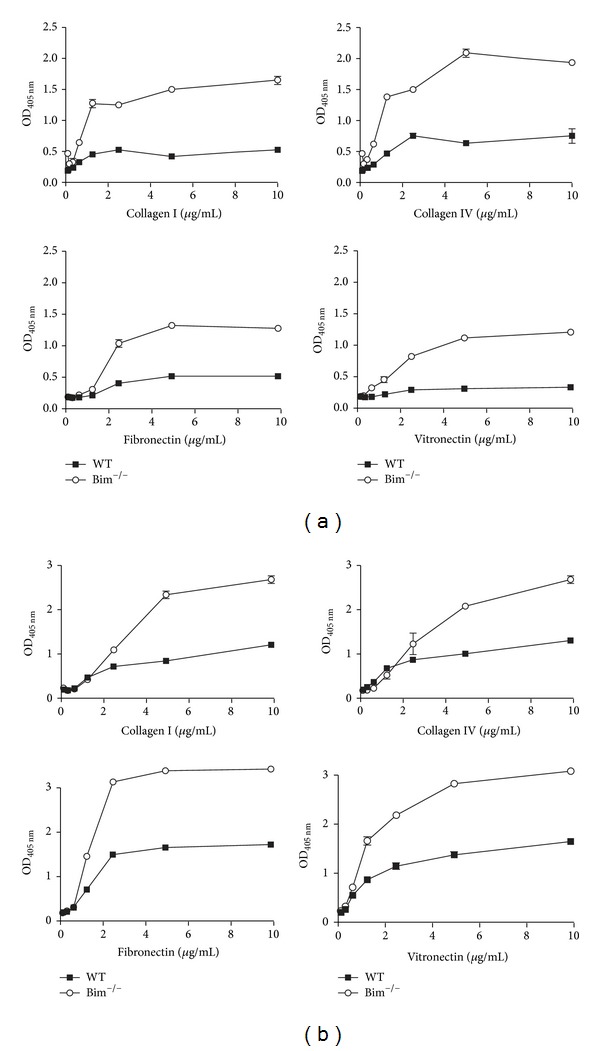
Bim^−/−^ endothelial cells and pericytes were more adherent. The adhesion of retinal wild-type (■) and Bim^−/−^ (○) endothelial cells (panel (a)) and pericytes (panel (b)) to fibronectin, vitronectin, collagen type I, or collagen IV was determined as described in Section 5. Please note that Bim^−/−^ vascular cells had increased adherence. These experiments were repeated twice with similar results.

Changes in migration of Bim^−/−^ retinal vascular cells could be due to altered cell adhesion. We next examined the ability of wild-type and Bim^−/−^ endothelial cells (Figure 4(a)) and pericytes (Figure 4(b)) to adhere to fibronectin, collagen I, collagen IV, and vitronectin. Bim deficient endothelial cells and pericytes displayed increased adhesion to all of these matrices compared to their wild-type counterpart. 

**Figure 5:**
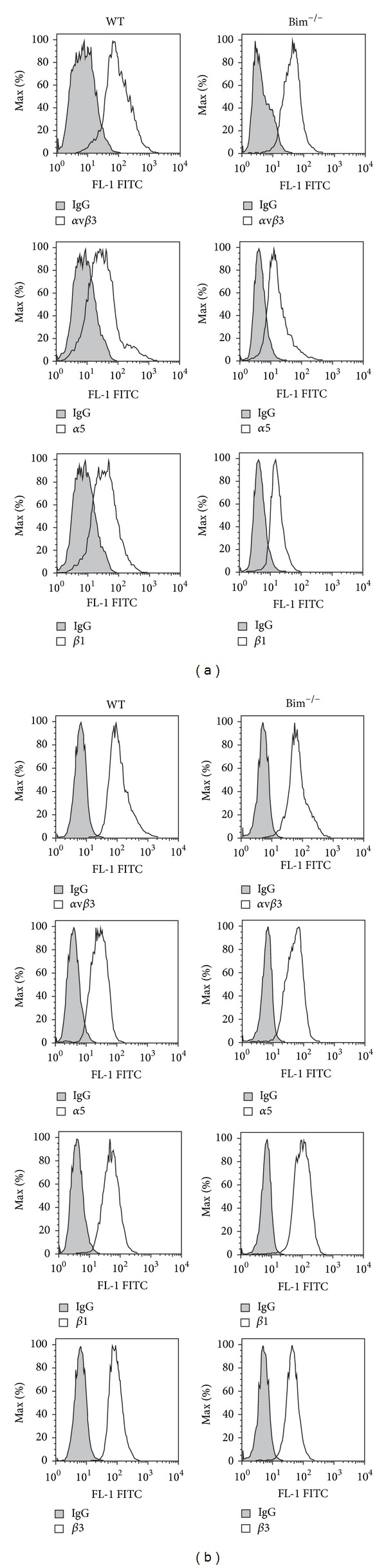
Wild-type and Bim^−/−^ retinal vascular cells had similar integrin expression. Expression of various integrins was determined in wild-type and Bim^−/−^ endothelial cells (panel (a)) and pericytes (panel (b)) using FACScan analysis as described in Section 5. The shaded graphs show staining in the presence of control IgG. These experiments were repeated twice with similar results.

Changes in cell migration and adhesion could be the result of aberrant integrin expression. Next we analyzed the integrin expression on the surface of retinal endothelial cells and pericytes by FACScan analysis (Figures 5(a) and 5(b)). Wild-type and Bim^−/−^ endothelial cells expressed similar levels of *α*5, *α*v*β*3, and *β*1 integrins on their surface while wild-type and Bim^−/−^ retinal pericytes expressed similar levels of *α*2, *α*v*β*3, *β*1, and *β*3  integrins. Thus, the increased adhesion noted in Bim^−/−^ retinal vascular cells may be independent of significant changes in the integrin expression and may be due to alterations in the affinity and/or avidity of these integrins.

### 2.4. Increased Tenascin C and Osteopontin Expression in Bim^−/−^ Retinal Endothelial Cells

**Figure 6:**
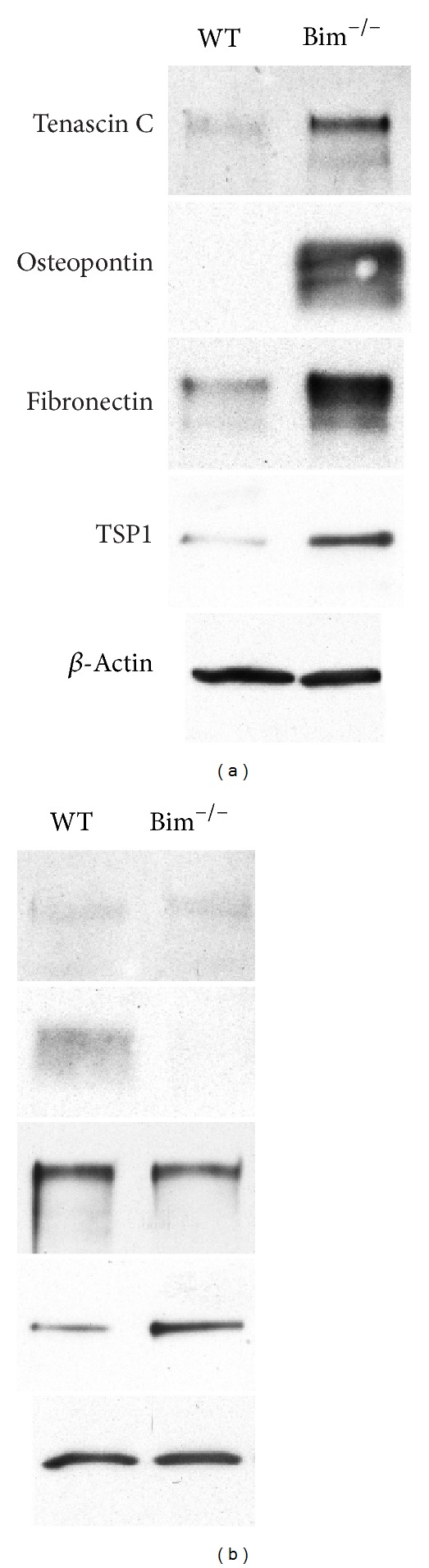
Altered expression of ECM proteins in Bim^−/−^ retinal cells. Wild-type and Bim^−/−^ retinal endothelial cells (panel (a)) and pericytes (panel (b)) were grown for 2 days in serum-free medium. The medium was harvested, clarified, and Western-blotted for extracellular matrix proteins as noted. The expression of *β*-actin from total cell lysates was used as a loading control. These experiments were repeated twice with similar results.

Modulating the ECM milieu can influence vascular cell functions including cell adhesion and migration. To examine whether the lack of Bim expression differentially impacts retinal endothelial cell or pericyte ECM expression, serum-free conditioned medium from these retinal vascular cells was evaluated by Western blot analysis. Retinal endothelial cells from Bim^−/−^ mice displayed an increased expression of tenascin C, osteopontin, fibronectin, and TSP1 compared to their wild-type counterpart (Figure 6(a)). In contrast, Bim^−/−^ retinal pericytes displayed similar levels of tenascin C, fibronectin, but an increased TSP1 and a decreased osteopontin expression (Figure 6(b)).

### 2.5. Decreased p-eNOS Expression in Bim^−/−^ Retinal Endothelial Cells

**Figure 7:**
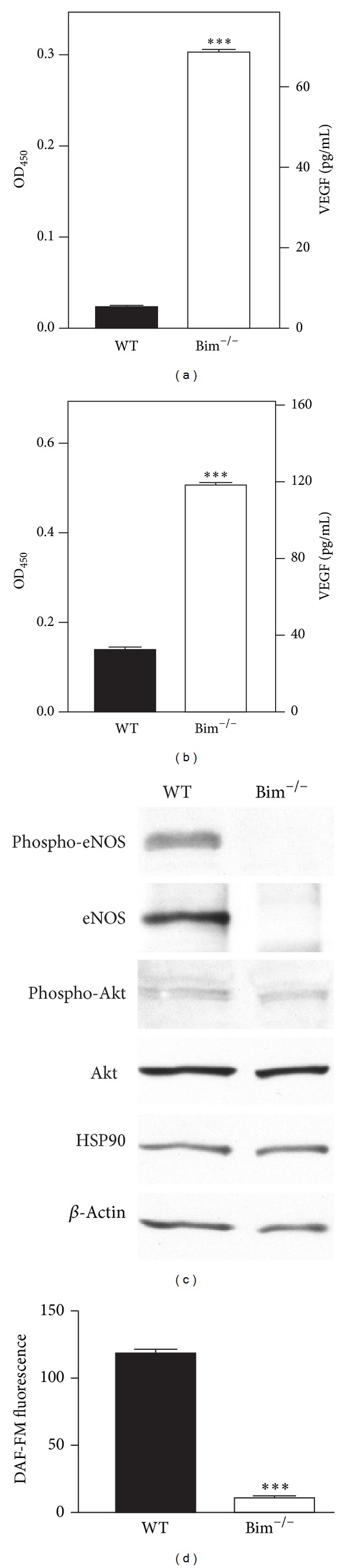
Decreased eNOS and increased VEGF expressions in Bim^−/−^ vascular cells. In panels (a) and (b), an immunoassay was used to determine VEGF levels (pg/mL) in retinal endothelial cells (a) and pericytes (b) from wild-type and Bim^−/−^ mice. Protein lysates (20 *μ*g) from wild-type and Bim^−/−^ retinal endothelial cells were analyzed by Western blot analysis for the expression of phospho-eNOS, eNOS, HSP90, phospho-Akt, and Akt (panel (c)). *β*-Actin expression was assessed as a loading control (panel (c)). In panel (d), the intracellular NO production was determined as analyzed by DAF-FM fluorescence for retinal endothelial cells. Please note that the increased VEGF expression was independent of eNOS expression in Bim^−/−^ endothelial cells (***P < 0.001).

The activation of eNOS and Akt1 by VEGF promotes angiogenesis [18–20]. In the absence of Bim, VEGF expression was dramatically increased in retinal endothelial cells and pericytes (Figures 7(a) and 7(b)). However, in Bim^−/−^ retinal endothelial cells, the increased VEGF expression did not affect Akt, phospho-Akt, or HSP90 expression (Figure 7(c)). Bim^−/−^ retinal endothelial cells also demonstrated nearly undetectable eNOS expression (Figure 7(c)) which corresponded with a 25-fold decrease in NO production (Figure 7(d)). Thus, VEGF activation of eNOS through the activation of Akt is uncoupled in the absence of Bim.

### 2.6. Bim^−/−^ Pericytes Disrupt Retinal Endothelial Cell Capillary Morphogenesis

Capillary morphogenesis is fundamental in vascular development and remodeling in which pericytes play a major role. The ability of pericytes to migrate and produce survival factors is central to their recruitment along the developing endothelium. However, pericytes typically do not undergo any substantial morphogenesis in Matrigel [21]. In the retina, endothelial cells and pericytes function together to facilitate retinal vascularization. Here, we evaluated the ability of pericytes from wild-type and Bim^−/−^ mice to impact retinal endothelial cell capillary morphogenesis.

**Figure 8:**
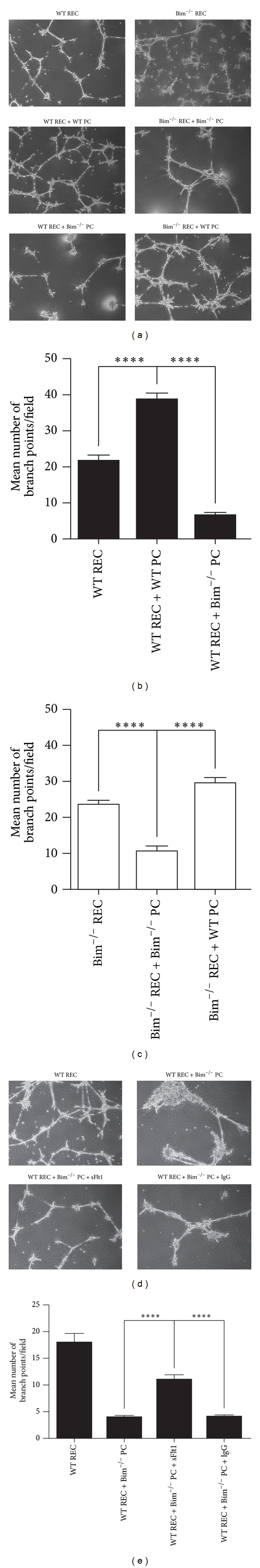
Bim^−/−^ pericytes diminished capillary morphogenesis of retinal endothelial cells. Wild-type (WT) and Bim^−/−^ retinal endothelial cells (REC) were plated alone or with wild-type or Bim^−/−^ pericytes (PC) in Matrigel and photographed in digital format (panel (a)). The quantitative assessment of the data is shown in panels (b) and (c). In panel (d), wild-type retinal endothelial cells were plated alone or with Bim^−/−^ pericytes in the presence or absence of sFlt1 or IgG control (10 *μ*g/mL) and photographed. Panel (e) is the quantitative assessment of the data in panel (d). The data are the mean number of branch points per field (×40) ± SD. These experiments were repeated at least twice with similar results (***P < 0.001; ****P < 0.0001).

Wild-type and Bim^−/−^ endothelial cells demonstrated a similar number of branch points in Matrigel (Figures 8(a)–8(c)). However, Bim^−/−^ endothelial cells formed rather stunted capillary-like tubes compared with wild-type cells (Figures 8(a)–8(c)). Next, we cocultured wild-type retinal endothelial cells with either wild-type or Bim^−/−^ pericytes. Wild-type retinal endothelial cells cocultured with wild-type pericytes demonstrated an improved tubular network compared to wild-type endothelial cells alone (Figures 8(a)–8(c)). In contrast, wild-type retinal endothelial cells cocultured with Bim^−/−^ pericytes demonstrated diminished capillary morphogenesis. Bim^−/−^ endothelial cells cocultured with wild-type pericytes demonstrated enhanced capillary morphogenesis while cocultures of Bim^−/−^ endothelial cells and pericytes demonstrated diminished capillary morphogenesis (Figures 8(a) and 8(c)). Thus, coculturing of retinal endothelial cells with Bim^−/−^ pericytes resulted in diminished endothelial cell capillary morphogenesis.

Bim^−/−^ pericytes demonstrated increased VEGF levels compared to their wild-type counterpart. Next, we addressed whether an increased VEGF expression contributed to the inability of wild-type endothelial cells to undergo capillary morphogenesis when cocultured with Bim^−/−^ pericytes. VEGFR-1 (Flt-1) has a higher affinity for VEGF compared to VEGFR-2 and can compete for VEGF binding to VEGFR-2 and its activation. Thus, the soluble form of Flt-1 can act as a trap for VEGF and prevent its signaling. Here, we used sFlt-1 FC chimera to demonstrate that the negative impact of Bim^−/−^ pericytes on capillary morphogenesis of retinal endothelial cells is driven by the production of excess VEGF. The sFlt1 was added to wild-type endothelial cell and Bim^−/−^ pericyte coculture experiments, and capillary morphogenesis was monitored (Figure 8(d)). As shown above, coculture of wild-type endothelial cells with Bim^−/−^ pericytes demonstrated decreased numbers of branch points compared to wild-type endothelial cells alone. The addition of sFlt1 to wild-type endothelial and Bim^−/−^ pericyte coculture experiments significantly restored branching morphogenesis in Matrigel. The addition of control IgG to wild-type endothelial cell and Bim^−/−^ pericyte coculture experiments did not impact the number of branch points (Figures 8(d) and 8(e)). Therefore, enhanced VEGF expression contributes to the decreased capillary morphogenesis of endothelial cells cocultured with Bim^−/−^ pericytes.

## 3. Discussion

Aberrant modulation of apoptosis has a reoccurring theme in many diseases that result in blindness. The regression of the hyaloid vasculature is an apoptosis-driven process in which Bim expression plays a central role [11]. Failure of the hyaloid vasculature to regress is a common congenital ocular malformation that can lead to blindness [22]. Improper modulation of apoptosis can also contribute to retinal vascular rarefaction and/or neovascularization, observed during retinopathy of prematurity and diabetic retinopathy. Thus, understanding how apoptosis is modulated in retinal vascular cells, including endothelial cells and pericytes, will further our understanding of normal and abnormal retinal vascularization and vascular cell autonomous functions.

The retina has the highest numbers of pericytes covering its blood vessels compared to other tissues [23]. The appropriate interaction of pericytes with each other and endothelial cells is important for vessel maturation and stabilization. Altered pericyte migration or adhesion may disrupt or enhance these interactions leading to vasculopathies or increased vascular stabilization. Here, we show that pericyte migration and adhesion are enhanced in the absence of Bim perhaps as a result of their increased VEGF expression. Increased pericyte migration may lead to greater pericyte coverage of the retinal vasculature, which may contribute to the precocious formation of the deep vascular plexus and protection from hyperoxia-mediated vessel obliteration that we previously observed [11].

Maturation of the retinal vasculature involves remodeling and pruning of vessels into an organized branched network of vessels to meet the demand of the tissue. Modulation of apoptosis by Bcl-2 family members facilitates retinal vascular remodeling and regression [11, 24]. Bim expression is required for retinal capillary pruning, hyaloid vessel regression, and hyperoxia-induced retinal vessel obliteration [11]. Here, we have extended these findings to delineate the role Bim plays in retinal endothelial cell and pericyte function. We show that in addition to enhanced migration, retinal pericytes from Bim^−/−^ mice were resistant to an apoptotic challenge and demonstrated an increased adhesion to extracellular matrices. Extracellular matrix protein expression by pericytes was only modestly impacted by the loss of Bim expression. Perhaps an increased pericyte adhesion in the absence of Bim enhances pericyte survival and coverage of retinal vessels. This may explain the resistance of the retinal vasculature of Bim^−/−^ mice to developmental pruning or excessive pruning in response to hyperoxia. Therefore, modulating pericyte adhesion and migratory properties may be essential for retinal vascular pruning.

VEGF expression is thought to be a key player in maintaining endothelial cell homeostasis. Since VEGF withdrawal can lead to endothelial cell apoptosis, determining how VEGF levels can be modulated by expression of Bcl-2 family members may aid in our understanding of normal and aberrant retinal vascularization and pruning. Here, we show that Bim deficiency increases VEGF levels in both endothelial cells and pericytes. Pericytes act as an angiogenic soup, producing 7-fold higher VEGF levels than their endothelial cell counterpart (Figure 7). In the absence of Bim, VEGF expression in retinal endothelial cells increased to nearly twice that observed in wild-type pericytes. Unfortunately, VEGF can also inhibit blood vessel maturation and ablate pericyte coverage of nascent sprouts causing vessel destabilization [25]. The increased VEGF produced by Bim^−/−^ pericytes may result in the diminished capillary morphogenesis observed when cocultured with either wild-type or Bim^−/−^ endothelial cells. Greenberg et al. [25] have also defined VEGF as an inhibitor of neovascularization which is consistent with the inability of Bim^−/−^ mice to undergo neovascularization following hyperoxia-induced ischemia [11]. However, retinal development in these mice proceeds quite well with precocious formation of the retinal vascular plexus and increased vascular density in contrast to what has been previously shown in fibrosarcomas [11, 25]. Thus, the ability of Bim to impact cell survival may be, in part, through the modulation of VEGF expression.

Phosphorylation of eNOS can mediate the proangiogenic activity of VEGF. Here, we show an increased VEGF expression but a decreased eNOS expression in Bim^−/−^ retinal endothelial cells, consistent with our previous studies in Bim^−/−^ kidney and lung endothelial cells [2]. Moreover, our recent studies further support a reciprocal relationship between VEGF and eNOS expressions in endothelial cells [26]. Our previous studies demonstrated that knocking down eNOS expression in kidney endothelial cells from diabetic mice not only decreased VEGF expression but also restored migration and capillary morphogenesis [26]. Thus, local regulation of Bim expression may play a central role in vascular pruning through modulating eNOS and VEGF expressions.

## 4. Conclusions

**Figure 9:**
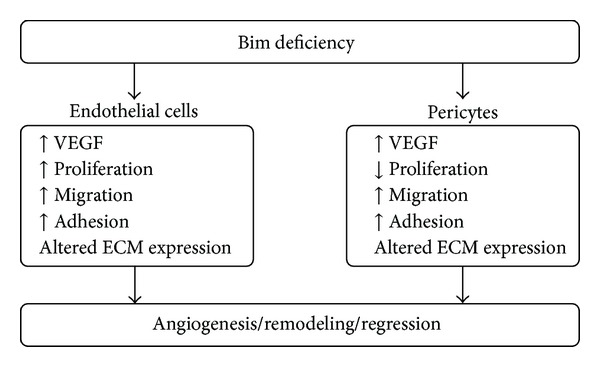
A summary of the modulation of VEGF expression, proliferation, migration, adhesion, and ECM expression in Bim deficient endothelial cells and pericytes.

Our previous studies demonstrated an inability of the retinas from Bim^−/−^ mice to undergo retinal vascular pruning leading to increased retinal cellularity and capillary loops [11]. These mice were also resistant to hyperoxia-mediated vessel obliteration and neovascularization during OIR, as well as a hyaloid vessel regression. Thus, Bim expression may modulate retinal vascular pruning by localized changes in retinal vascular cell adhesion, migration, and VEGF expression (Figure 9) thereby, either protecting or inducing pruning by localized apoptosis.

## 5. Methods

### 5.1. Experimental Animals and Cell Cultures

The mice used for these studies were maintained and treated in accordance with our protocol approved by the University of Wisconsin Animal Care and Use Committee. Immortomice expressing a temperature-sensitive SV40 large T antigen were obtained from Charles River Laboratories (Wilmington, MA, USA). Bim^−/−^ mice (Jackson Laboratory, Bar Harbor, ME, USA) were crossed with Immortomice and screened as previously described [2, 17]. To isolate retinal endothelial cells, retinas from approximately four- to five-week-old wild type and Bim^−/−^ Immortomice were dissected out aseptically and placed in serum-free Dulbecco's Modified Eagle Medium (DMEM) containing penicillin/streptomycin (Sigma, St. Louis, MO, USA). The retinas were pooled together, rinsed with DMEM, minced into small pieces in a 60 mm tissue culture dish using sterilized razor blades, and digested in 5 mL of collagenase type I (1 mg/mL in serum-free DMEM; Worthington, Lakewood, NJ, USA) for 30–45 minutes at 37°C. Following digestion, DMEM containing 10% fetal bovine serum (FBS) was added and cells were pelleted. The cellular digests were then filtered through a double layer of sterile 40 *μ*m nylon mesh (Sefar America Inc., Hanover Park, IL, USA), centrifuged at 400 ×g for 10 min to pellet cells, and then washed twice with DMEM containing 10% FBS. The cells were resuspended in 1.5 mL medium (DMEM with 10% FBS) and incubated with sheep anti-rat magnetic beads precoated with anti-PECAM-1 antibody (MEC13.3, BD Biosciences, Bedford, MA, USA) as described previously [27]. After affinity binding, magnetic beads were washed six times with DMEM containing 10% FBS. Bound cells in endothelial cell growth medium were plated into a single well of a 24-well plate precoated with 2 *μ*g/mL of human fibronectin (BD Biosciences). Endothelial cells were grown in DMEM containing 10% FBS, 2 mM L-glutamine, 2 mM sodium pyruvate, 20 mM HEPES, 1% nonessential amino acids, 100 *μ*g/mL streptomycin, 100 U/mL penicillin, heparin at 55 U/mL (Sigma, St. Louis, MO, USA), endothelial growth supplement 100 *μ*g/mL (Sigma, St. Louis, MO, USA), and murine recombinant interferon-*γ* (R&D, Minneapolis, MN, USA) at 44 units/mL. Cells were maintained at 33°C with 5% CO_2_. Cells were progressively passed to larger plates, maintained, and propagated in 1% gelatin-coated 60 mm dishes. Retinal endothelial cells were positive for B4-lectin (a mouse endothelial cell specific lectin) and expressed PECAM-1 and VE-cadherin as previously described [2, 14].

Pericytes were isolated from mouse retina by collecting retinas from one litter (6-7 pups, 4 weeks old) using a dissecting microscope. Retinas (12 to 14) were rinsed with serum-free Dulbecco's Modified Eagle's Medium (Sigma), pooled in a 60 mm dish, minced, and digested for 45 min with collagenase type II (1 mg/mL; Worthington, Lakewood, NJ, USA) with 0.1% bovine serum albumin (BSA) in serum-free DMEM at 37°C. Cells were rinsed in DMEM containing 10% fetal bovine serum (FBS) and centrifuged for 5 min at 400 ×g. Digested tissue was resuspended in 4 mL of DMEM containing 10% FBS, 2 mM L-glutamine, 100 *μ*g/mL streptomycin, 100 U/mL penicillin, and the murine recombinant interferon-*γ* (R&D, Minneapolis, MN, USA) at 44 U/mL. Cells, tissue, and medium were evenly divided into 4 wells of a 24-well tissue culture plate and maintained at 33°C with 5% CO_2_. Cells were progressively passed to larger plates, maintained, and propagated in 60 mm dishes [21]. The experiments described here were performed with two separate isolations of cells with similar results.

### 5.2. Cell Apoptosis Assays

Apoptosis was determined by measuring caspase activation using a Caspase-Glo 3/7-assay kit as recommended by the supplier (Promega, Madison, WI, USA). The assay provides caspase-3/7 DEVD-aminoluciferin substrate and the caspase 3/7 activity is detected by luminescent signal. For the assay, cells were plated at 8 × 10^3^ per well of a 96-well plate. As an oxidative or apoptotic stimulus, cells were incubated with 10 nM staurosporine (Invitrogen), in growth medium for 24 h at 33°C. Caspase activity was detected using a luminescent microplate reader (Victa2 1420 Multilabel Counter, PerkinElmer, Waltham, MA, USA). All samples were prepared in triplicate and repeated twice with similar results [15].

### 5.3. Scratch Wound Assay

Cells (4 × 10^5^) were plated in 60 mm tissue culture dishes and allowed to reach confluence (2-3 days). After aspirating the medium, cell layers were wounded using a 1 mL micropipette tip. Plates were then rinsed with PBS, fed with growth medium containing 100 ng/mL of 5-fluorouracil (5-FU), to rule out potential contribution of differences in cell proliferation. The wounds were observed and photographed every 24 hours for up to 72 hours. The distance migrated was determined as a percent of total distance for quantitative assessments as described previously [2]. These experiments were repeated at least twice with two different isolations with similar results.

### 5.4. Capillary Morphogenesis in Matrigel

Matrigel (10 mg/mL; BD Biosciences) was applied at 0.5 mL/well of a 6-well tissue culture dish and incubated at 37°C for at least 30 minutes to harden. Cells were removed using trypsin-EDTA, washed with growth medium once, and resuspended at 1 × 10^5^ cells per mL in serum-free growth medium. For coculture experiments, retinal endothelial cells and pericytes were used at a 1 : 1 ratio as we previously described [28]. Cells (2 mL) were gently added to the Matrigel coated plates, incubated at 37°C, monitored at ~18 h, and photographed using a Nikon microscope equipped with a digital camera. In some cases, 10 *μ*g/mL of control IgG or sFlt1-FC chimera (R&D Systems) was added to the coculture experiments. For a quantitative assessment of the data the mean number of branch points in 8 fields (×40) was determined. A longer incubation of the cells did not result in further branching morphogenesis [27].

### 5.5. Cell Adhesion Assays

Cell adhesion to various extracellular matrix proteins was performed as previously described [9]. Varying concentrations of fibronectin, human type I and IV collagen, and vitronectin (BD Biosciences) prepared in TBS with Ca^2+^Mg^2+^ (2 mM each; TBS with Ca^2+^Mg^2+^) were coated on 96-well plates (50 *μ*L per well; Nunc MaxiSorp plates, Fisher Scientific) overnight at 4°C. Plates were then rinsed four times with TBS with Ca^2+^Mg^2+^ and blocked with 200 *μ*L of 1% BSA prepared in TBS with Ca^2+^Mg^2+^ for at least 1 h at room temperature. Cells were removed using 1.5 mL dissociation solution, washed once with TBS, and resuspended at 5 × 10^5^ cells/mL in HEPES-buffered saline (25 mM HEPES, pH 7.60, 150 mM NaCl, and 4 mg/mL BSA). After blocking, plates were rinsed with TBS Ca^2+^Mg^2+^ once, 50 *μ*L of cell suspension was added to each well containing 50 *μ*L of TBS with Ca^2+^Mg^2+^, and the cells were allowed to adhere for 90 min at 37°C in a humidified incubator. Nonadherent cells were removed by gently washing the plate four times with 200 *μ*L of TBS with Ca^2+^Mg^2+^ until no cells were left in wells coated with BSA. The number of adherent cells in each well was quantified by measuring the levels of intracellular acid phosphatase. Cells were lysed in 100 *μ*L of lysis buffer (50 mM sodium acetate pH 5.0, 1% Triton X-100, and 4 mg/mL p-nitrophenyl phosphate) and incubated at 4°C overnight. The reaction was neutralized by adding 50 *μ*L of 1 M NaOH, and the absorbance was determined at 405 nm using a microplate reader (Thermomax, Molecular Devices). All samples were prepared in triplicate, and the experiments were repeated at least three times with similar results.

### 5.6. Western Blot Analysis

Cells were plated at 4 × 10^5^ in 60 mm dishes coated with 1% gelatin (endothelial cells) or uncoated (pericytes) and allowed to reach nearly 90% confluence in 2 days. The cells were then rinsed once with serum-free medium and incubated with serum-free DMEM for 48 hours. Then, conditioned medium (2 mL) was collected and clarified by centrifugation. The 45 *μ*L of the sample was mixed with appropriate volume of 6X SDS buffer and analyzed by SDS-PAGE (4–20% Tris glycine gel; Invitrogen, Carlsbad, CA). In some cases, total protein lysates were prepared from these cells in a modified RIPA buffer (142.5 mM KCl, 5 mM MgCl_2_, 10 mM HEPES, pH 7.4, 2 mM orthovanadate and 2 mM sodium difluoride, 1% Nonidet P-40, and a complete protease inhibitor cocktail (Roche, Mannheim, Germany)). The proteins were transferred to a nitrocellulose membrane, and the membrane was incubated with an antifibronectin (Sigma), a rabbit anti-chicken tenascin-C polyclonal antibody (Thermo Scientific; Pierce, Rockford, IL, USA), anti-TSP1 monoclonal antibody (Clone A6.1, Neo Marker, Fremont, CA, USA), antiosteopontin (R&D, Minneapolis, MN), anti-*β*-actin (Thermo Scientific; Pierce, USA), anti-HSP90 (Cell Signaling Technology), anti-Akt (Cell Signaling) antiphospho-Akt (Cell Signaling), antiphospho-eNOS (Cell Signaling), and anti-eNOS (SC-654; Santa Cruz Technology, Santa Cruz, CA). Membranes were washed, incubated with horseradish-peroxidase-conjugated secondary antibody (1 : 5000, Jackson ImmunoResearch Laboratories, West Grove, PA) for 1 hour at room temperature, and the protein was visualized according to the chemiluminescent procedure (Chemiluminescence reagent; GE Biosciences) [2, 14].

### 5.7. Flow Cytometry

Flow cytometry was performed as previously described [14]. The cells were washed once with PBS containing 0.04% EDTA and incubated with 2 mL of dissociation solution (Sigma) to remove the cells from the plate. The cells (10^6^) were washed with TBS, blocked in TBS containing 1% goat serum on ice for 20 minutes, and incubated with the appropriate dilution of primary antibody, anti-PECAM-1 (BD Pharmingen), antivascular endothelial (VE)-cadherin (Alexis Biochemical, San Diego, CA), anti-*β*1 (Millipore), anti-*α*5 (MAB1949; Millipore), anti-*α*2 (Chemicon), anti-*β*3 (MAB1957; Millipore), anti-*α*v*β*3 (MAB1976Z; Millipore), control IgG (Jackson), rabbit anti-NG2 (AB5320; Millipore, Temecula, CA), and rat anti-mouse PDGFR*β* (eBiosciences, San Diego, CA). For antibodies that required cell permeabilization, cells were removed, washed with PBS, fixed with 2% paraformaldehyde on ice for 30 min, washed with PBS, and resuspended in PBS containing 0.1% Triton-X-100 and 0.1% BSA containing appropriate dilution of primary antibody. The cells were washed with TBS containing 1% BSA and then incubated with the appropriate FITC-conjugated secondary antibody (Jackson ImmunoResearch, West Grove, PA) for 30 min on ice. After the incubation, the cells were washed twice with TBS containing 1% BSA and resuspended in 0.5 mL of TBS containing 1% BSA. Analysis was performed on a FACScan caliber flow cytometer (Becton-Dickinson, Franklin Lakes, NJ).

### 5.8. Transwell Assay

Transwell filters (Corning, Acton, MA) were coated with 2 *μ*g/mL fibronectin in PBS and incubated overnight at 4°C. The bottom of the transwell was rinsed with PBS and blocked with 2% BSA in PBS for 1 h at room temperature. The transwell was rinsed with PBS, and 500 *μ*L serum-free DMEM was added to the bottom of each well, and 1 × 10^5^ cells in 100 *μ*L of serum-free medium were added to the top of the transwell membrane. Following 4 hours in a 33°C tissue culture incubator, the cells and medium were aspirated and the upper side of the membrane was wiped with a cotton swab. The cells that migrated through the membrane were fixed with 4% paraformaldehyde, stained with hematoxylin-eosin, and mounted on a slide. Ten-high power fields (×200) of cells were counted for each condition and the average and standard error of the means were determined. All samples were prepared in duplicate, and the experiment was repeated at least three times with similar results.

### 5.9. VEGF Analysis

VEGF protein levels produced by vascular cells were determined using a Mouse VEGF Immunoassay kit (R&D Systems, Minneapolis, MN). Cells were plated at 6 × 10^5^ cells on 60 mm tissue culture dishes and allowed to reach approximately 90% confluence. The cells were then rinsed once with serum-free DMEM and were grown in serum-free medium for 2 days. Conditioned medium was centrifuged at 400 ×g for 5 min to remove cell debris, and 50 *μ*L was used in the VEGF Immunoassay. The assay was performed in triplicate as recommended by the manufacturer and was normalized to the number of cells. The amount of VEGF was determined using a standard curve generated with known amounts of VEGF in the same experiment. The assay was repeated twice using two different isolations of endothelial cells with similar results.

### 5.10. Nitric Oxide Analysis

Cells were plated in black wall clear bottom Microtest 96-well plates (BD #35 3948; 1 × 10^4^ cells in 100 *μ*L). The next morning the medium was changed to endothelial cell or pericyte medium containing 30 *μ*M DAF-FM diacetate (Invitrogen; D-23842) and 5 *μ*g/mL of CellTracker Red (Invitrogen; C34552). Following a 40 min incubation at 33°C, fresh medium was placed on the cells and the incubation continued for 20 min. The wells were washed with TBS and the cells in each well were resuspended in 100 *μ*L of TBS. Absorbance was read at 495/515 nm using a fluorescence plate reader [2]. These experiments were performed in triplicate and repeated twice with similar results.

### 5.11. Statistical Analysis

Statistical differences between groups were evaluated with an unpaired *t*-test (two-tailed). Mean ± standard deviations are shown. *P* values <0.05 were considered significant.
